# High Heterogeneity of Echoviruses in Brazilian Children with Acute Gastroenteritis

**DOI:** 10.3390/v13040595

**Published:** 2021-03-31

**Authors:** Endrya do Socorro Fôro Ramos, Ulisses Alves Rosa, Geovani de Oliveira Ribeiro, Fabiola Villanova, Flávio Augusto de Pádua Milagres, Rafael Brustulin, Vanessa dos Santos Morais, Mayara Bertanhe, Roberta Marcatti, Emerson Luiz Lima Araújo, Steven S. Witkin, Eric Delwart, Adriana Luchs, Antonio Charlys da Costa, Élcio Leal

**Affiliations:** 1 Laboratório de Diversidade Viral, Instituto de Ciências Biológicas, Universidade Federal do Pará, Belem 66075-000, Pará, Brazil; endrya.ramos@gmail.com (E.d.S.F.R.); ualvesfisio@yahoo.com.br (U.A.R.); geovanibiotec@gmail.com (G.d.O.R.); fvillanova@gmail.com (F.V.); 2 Secretaria de Saúde do Tocantins, Palmas 77453-000, Tocantins, Brazil; flaviomilagres@uft.edu.br (F.A.d.P.M.); eu3rafael@gmail.com (R.B.); 3 Laboratório Central de Saúde Pública do Tocantins (LACEN/TO), Palmas 77016-330, Tocantins, Brazil; 4 Departamento de Moléstias Infecciosas e Parasitárias, Instituto de Medicina Tropical, Faculdade de Medicina, Universidade de São Paulo, São Paulo 05403-000, Brazil; va.morais@usp.br (V.d.S.M.); mayarabertanhe@globo.com (M.B.); robertamarcatti@gmail.com (R.M.); switkin@med.cornell.edu (S.S.W.); 5 General Coordination of Public Health Laboratories of the Strategic Articulation, Department of the Health Surveillance Secretariat of the Ministry of Health (CGLAB/DAEVS/SVS-MS), Brasília 70719-040, Federal District, Brazil; emerson.araujo@saude.gov.br; 6 Department of Obstetrics and Gynecology, Weill Cornell Medicine, 1300 York Avenue, New York, NY 10065, USA; 7 Vitalant Research Institute, 270 Masonic Avenue, San Francisco, CA 94143, USA; EDelwart@Vitalant.org; 8 Department Laboratory Medicine, University of California San Francisco, San Francisco, CA 94143, USA; 9 Laboratório de Doenças Entéricas, Centro de Virologia, Instituto Adolfo Lutz, São Paulo 01246-000, Brazil

**Keywords:** echovirus E, acute gastroenteritis, enterovirus, gut virome, pediatric virome

## Abstract

Echoviruses (E) are a diverse group of viruses responsible for various pathological conditions in humans including aseptic meningitis, myocarditis, and acute flaccid paralysis. The detection and identification of echovirus genotypes in clinical samples is challenging due to its high genetic diversity. Here, we report the complete genome sequences of nine echoviruses, obtained by next-generation sequencing of 238 fecal samples from individuals with gastroenteritis in regions of Brazil. Detected viruses were classified into six genotypes: Three E1 sequences (BRA/TO-028, BRA/TO-069 and BRA/TO-236), one E3 (BRA/TO-018), one E11 (BRA/TO-086), one E20 (BRA/TO-016), two E29 (BRA/TO-030 and BRA/TO-193), and one E30 sequence (BRA/TO-032). Phylogenetic analysis indicated that the echoviruses E1 and E29 circulating in Brazil are divergent from strains circulating worldwide. The genotype diversity identified in our study may under-represent the total echovirus diversity in Brazil because of the small sample size and the restricted geographical distribution covered by the survey.

## 1. Introduction

Echovirus (enteric cytopathic human orphan virus) is a polyphyletic group of viruses associated with enteric disease in humans [1]. The echovirus is group of single strand positive-sense RNA virus belonging to species B within the genus *Enterovirus* (EVs) of the family *Picornaviridae* [1]. These viruses were originally not associated with disease, but many have since been identified as disease-causing agents. It was described first in 1950 from patients with aseptic meningitis as well as in healthy children [2]. Subsequently, in several studies worldwide, its occurrence was associated with a myriad of clinical manifestations, including aseptic meningitis, acute flaccid paralysis, encephalitis, myocarditis, and neonatal sepsis as well as being present in asymptomatic individuals [3,4,5,6,7].

The echovirus genome is similar to other EV that contain approximately 7.4–7.5 Kb. a 5′ UTR region, followed by a single open reading frame (ORF) and then by a 3′ UTR region. The ORF is translated and cleaved into three polyprotein precursors: P1, P2, and P3 that encode four structural proteins (VP1–VP4: P1 region) and seven non-structural proteins (2A–2C: region P2 and 3A–3D: region P3) [8,9]. The VP1 protein is the most widely studied since it is the most variable coding region of the genome and is used to classify EVs into genotypes [8,9,10].

At the present time, echovirus comprises 28 genotypes (E1 to E7, E9, E11 to E21, E24 to E27,E29 to E33) [1,11] and is widely detected [7,12,13,14,15] including in Brazil [6,15,16,17,18,19,20]. Brazil is a country with continental dimensions that spans from tropical to temperate latitudes, experiencing variable climatic conditions and geographically distinct populations. Therefore, a high degree of echovirus diversity would be expected in this country. Continuous multiregional surveillance is vital to establish a more complete epidemiological molecular picture of the echovirus burden in Brazil [16]. However, detection and identification of echovirus genotypes in clinical samples is challenging due to its high genetic diversity [8]. Recently, next-generation sequencing (NGS) has been providing a new method for identifying echovirus [21,22].

In this study, we report the complete genome sequences of nine echovirus sequences encompassing six genotypes (E1, E3, E11, E20, E29 and E30) obtained during a NGS surveillance conducted on children suffering from acute gastroenteritis in the Northern region of Brazil. Phylogenetic analysis was performed for comparison with other previously reported strains.

## 2. Materials and Methods

### 2.1. Ethical Aspects

The survey was conducted in accordance with the 1975 Helsinki Declaration (https://www.wma.net/what-we-do/medical-ethics-of-helsinki/, accessed on 5 February 2021), revised in 2013. The project was approved by the Ethics Committee of the institutions involved (Faculty of Medicine of the University of São Paulo (CAAE: 53153916.7.0000.0065), and Centro Universitário Luterano de Palmas-ULBRA (CAAE: 53153916.7.3007.5516). There was no risk or harm to the children or their guardians, so it was not necessary to apply the Free and Informed Consent Term (ICF) in accordance with resolution 196/96 on research involving human beings—National Health Council (CNS)/Ministry of Health (MS), Brasília, 1996.

### 2.2. Study Population and Specimen Collection

The current cross-sectional surveillance study was carried out from 2010 to 2016 in the states of Tocantins (TO), Maranhão (MA), and Pará (PA), two regions in the North (TO/PA) and Northeast (MA) of Brazil. Fecal samples were collected at 36 different sites. In total, 238 specimens were obtained, 232 from the state of Tocantins, and 3 from the state of Pará. Three samples were obtained in border municipalities (Estreito and Carolina) located between Tocantins and Pará. A total of 94 stool samples were obtained from children under 1 year old, 139 stool samples were from children 1 to 5 years old, and 5 stool samples were from 6 to 12 years old with symptoms of gastroenteritis.

### 2.3. Sample Screening

All samples were initially sent to the Public Health Laboratory of Tocantins (LACEN-TO), accompanied by an epidemiological investigation record including demographic data (i.e., age, gender, and date of collection) and clinical information (i.e., signs and symptoms) of the participants. LACEN-TO conducted the identification for common enteric pathogens such as bacteria (i.e., *Escherichia coli* and *Salmonella* sp.) and parasites (i.e., *Giardia* sp. and *Taenia solium*), using conventional cultivation techniques and parasitological tests, such as Hoffman’s method and fresh direct examination. The samples were stored at −20 °C and forwarded to the USP Institute of Tropical Medicine of the University of São Paulo (IMT-USP) for identification of gastroenteric viruses (rotavirus, norovirus, human adenovirus, human astrovirus, human bocavirus, and human sapovirus) [23,24,25,26].

### 2.4. Viral Metagenomics

The protocol used to perform deep sequencing was a combination of several protocols applied to viral metagenomics and/or virus discovery, according to procedures previously described [27]. In summary, 50 mg of each human fecal sample was diluted in 500 μL of Hank’s buffered saline (HBSS) and added to a 2 mL impact resistant tube containing C lysing matrix (MP Biomedicals, Santa Ana, CA, USA) and homogenized in a FastPrep-24 5G homogenizer (MP biomedicals). The homogenized sample was centrifuged at 12,000× *g* for 10 min and approximately 300 μL of the supernatant was then percolated through a 0.45 μm filter (Merck Millipore, Billerica, MA, USA) to remove bacterial and eukaryotic cells. Approximately 100 μL of PEG-it Virus Precipitation solution (System Biosciences, Palo Alto, CA, USA) was added to the filtrate and the contents of the tube were gently homogenized followed by incubation at 4 °C for 24 h. After the incubation period, the mixture was centrifuged at 10,000× *g* for 30 min at 4 °C and the supernatant (~350 μL) was discarded. The granulate, rich in viral particles, was treated with a combination of nuclease enzymes (TURBO DNase and RNase Cocktail Enzyme Mix-Thermo Fischer Scientific, Waltham, MA, USA; Baseline-ZERO DNase DNase-Epicenter, Madison, WI, USA; Benzonase -Darmstadt, Darmstadt, Germany and RQ1 DNase-Free DNase and RNase A Solution-Promega, Madison, WI, USA) to digest unprotected nucleic acids. The resulting mixture was subsequently incubated at 37 °C for 2 h. The viral nucleic acids were then extracted using the ZR & ZR-96 viral DNA/RNA kit (Zymo Research, Irvine, CA, USA), according to the manufacturer’s instructions. The synthesis of cDNA was performed with an AMV reverse transcription reagent (Promega, Madison, WI, USA). A second-strand cDNA synthesis was performed using a large DNA polymerase I fragment (Klenow) (Promega, Madison, WI, USA). Subsequently, a Nextera XT Sample Preparation Kit (Illumina, San Diego, CA, USA) was used to build a DNA library, which was identified using double barcodes. The library was then purified using the ProNex^®^ size selective purification system (Promega, WI, USA). Following the ProNex^®^ purification, the quantity of each sample was normalized to ensure an equal representation of the library with the combined samples using the ProNex^®^ NGS Library Quant Kit (Promega). For size range, Pippin Prep (Sage Science, Inc.) was used to select a 300 bp tablet (range 200 to 400 bp), which excluded very short and long fragments from the library. Before the generation of the cluster, the libraries were quantified again by qPCR using the ProNex^®^ NGS Library Quant Kit (Promega). The library was sequenced in depth using a Hi-Seq. 2500 sequencer (Illumina) with ends of 126 bp [27,28,29].

Bioinformatics analysis was performed according to the protocol previously described by Deng et al. [30]. Contigs, including sequences of enteric viruses, fungi, bacteria, and others, sharing a percentage nucleotide identity of 95% or less, were assembled from the sequence readings obtained by de-assembly. The resulting singlets and contigs were analyzed using BLASTx to look for similarity with viral proteins on GenBank. The contigs were compared with GenBank’s non-redundant nucleotide and protein databases (BLASTn and BLASTx). After virus identification, a reference model sequence was used to map the complete genome with the Geneious R9 software (Biomatters Ltd. L2, 18 Shortland Street, Auckland, 1010, Auckland, New Zealand).

### 2.5. Molecular Typing of Echovirus

To assign genotypes to the strains detected in the present study, the publicly available Enterovirus Genotyping Tool (https://www.rivm.nl/mpf/typingtool/enterovirus, accessed on 5 February 2021) [31] was used in order to assign genotypes and to confirm previous BLASTx classification. We confirmed the previous classification into E1 (BRA/TO-028, BRA/TO-069 and BRA/TO-236), E3 (BRA/TO-018), E11 (BRA/TO-086), E20 (BRA/TO-016), E29 (BRA/TO-030, BRA/TO-193) and E30 (BRA/TO-032) obtained through a BLASTx search.

### 2.6. Alignments and Phylogenetic Analysis

The full genome sequences obtained in the study and a set of cognate sequences (see the list of sequences in Table S1 in the supplementary material) available on GenBank were aligned using the MUSCLE algorithm [32] implemented in Molecular Evolutionary Genetics Analysis software program MEGA v.5 [33]. Phylogenetic analysis was performed based on the maximum likelihood approach with bootstrap test setting at 1000 consensus replicates to obtain reproducible results and provide greater reliability to the clusters. The support values of the branches were evaluated by the Shimodaira-Hasegawa test. All trees were inferred using the FastTree [34] software and the GTR model with gamma distribution was selected according to the likelihood ratio test (LRT) implemented in the jModeltest [35] software. Likelihood mapping was obtained using the software Tree-puzzle, version 5.3 [36], assuming the GTR model and rate of heterogeneity for the evolutionary model. Analyses were performed using 1000 replications.

### 2.7. Evolutionary Distances

Genetic distance and their standard error were calculated using the maximum composite likelihood model plus gamma correction and bootstrap with 100 replicates. Distances were calculated using MEGA software (Version X) [33]. To estimate the similarity of sequences we used a pair-wise method implemented in the program SDT [37]. To estimate the similarity, alignments of every unique pair of sequences were done using algorithms implemented in Mafft [38]. After the computation of the identity score for each pair of sequences, the program then uses the NEIGHBOR component of PHYLIP to compute a tree [39]. The rooted neighbor-joining phylogenetic tree orders all sequences according to their likely degrees of evolutionary relatedness. Results are presented in a frequency distribution of pairwise-identities in a graphical interface and sequences are ordered according to their similarity.

### 2.8. Recombination Analysis

We used RDP v.4 software [40], which utilizes a collection of methods, to determine the extent of recombination among sequences. Below is a brief description of these methods, and an excellent and detailed explanation of each method implemented in the RDP program can be found in the user’s manual (Available online: http://darwin.uvigo.es/rdp/rdp.html, accessed on 5 February 2021). Maximum χ2 (MaxChi) is a method, implemented by Maynard Smith, that uses variable/invariable sites to detect recombination in pairs of sequences. This method generates random sequence pairs. The significance level is evaluated by the proportion of simulated sequence pairs with maximum χ2 values higher than the real data. The maximum match χ2 (Chimaera) is a modification of Smith’s method. It uses variable sites to calculate the maximum χ2 match statistics. Geneconv detects gene conversions (recombination) by evaluating conserved substitutions in fragments between two sequences. Although evolutionary methods are not explicitly implemented in Geneconv, it is robust and has low levels of false positive detection of recombination, including those events due to rate heterogeneity and natural selection. Lard is similar to MaxChi and the method scans an alignment of three sequences (a recombinant and two parental sequences) for the point in the alignment that optimally separates regions of conflicting phylogenetic signals; p-values are also estimated to the breakpoint. Initially, we used default parameters. We later optimized the parameters to avoid detection of false positive recombination. In addition, window sizes of 50 to 350, stepping of 50–100 nt, as well as Bonferroni correction with p-values of 0.05 and 0.001 were utilized. RDP uses the program INTERVAL from the LDHAT package [41] to construct plots of varying recombination rates across sequences. INTERVAL estimates site-by-site variations in recombination rates (rho) along the lengths of nucleotide sequence alignments. The approach calculates variations in recombination rates along an alignment using a penalized approximate likelihood approach within a Bayesian reversible-jump Markov chain Monte Carlo (RJMCMC) scheme.

### 2.9. Accession Numbers

Nucleotide sequences determined in this study have been deposited in GenBank under the accession number: MW775340 to MW775348.

## 3. Results

### 3.1. Echovirus Identification

**Figure 1:**
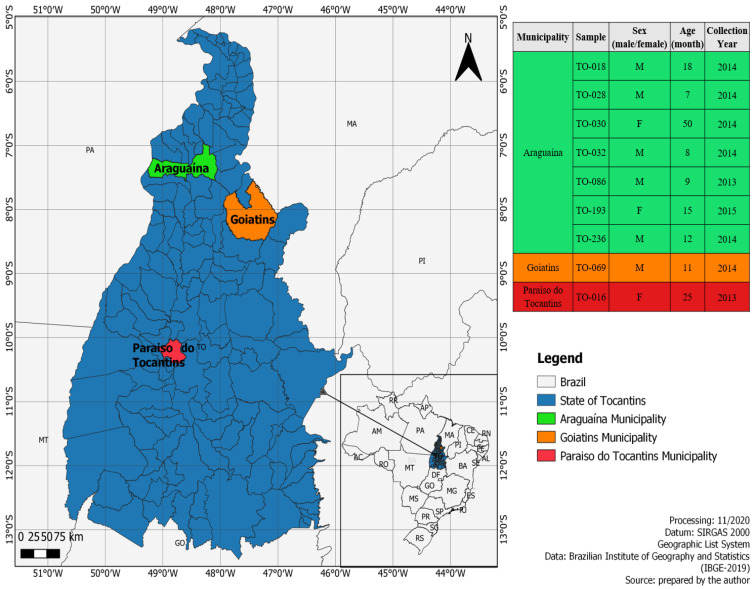
Map of the State of Tocantins (Northern, Brazil) highlighting municipalities (in red, green, and orange) where the positive echovirus fecal samples were collected. Upper figure: Positive localities in the state of Tocantins. Lower figure: Map of Brazil showing the state of Tocantins area. The map was generated with the software QGIS Geographic Information System.

The viral sequences obtained were identified utilizing sequence identity through BLASTx for viral genomes annotated on GenBank. The search returned nine sequences homologous to other echovirus strains available in the database. Children were residents in urban areas of Araguaína, Goiatins and Paraiso do Tocantins municipalities (state of Tocantins), and were experiencing acute gastroenteritis symptoms such as diarrhea, vomiting, and fever (Figure 1, Table 1).

### 3.2. Echovirus Genotyping

The genotypes of the Echovirus sequences identified in the present investigation were assigned according to the highest identity score in the BLAST Program and confirmed using the Enterovirus Genotyping Tool. Nucleotide identity values obtained in the BLAST search ranged from 77% to 83%.

The nine echovirus sequences identified were classified into six genotypes: Three E1 (BRA/TO-028, BRA/TO-069 and BRA/TO-236), one E3 (BRA/TO-018), one E11 (BRA/TO-086), one E20 (BRA/TO-016), two E29 (BRA/TO-030 and BRA/TO-193), and one E30 (BRA/TO-032) (Table 1).

### 3.3. Genetic Distances and Phylogenetic Inferences

Estimates of pair-wise differences using near-full length genomes showed that the Brazilian E20 sequence (TO-016/BRA) has 19.30% of genetic distance at the nucleotide level (nt) compared to the AY302546_USA/1956 prototype strain, and 19.30–20.21% when compared to representative E20 group detected in Haiti in 2016, China in 2010, and Nigeria in 2010 and 2014. The three Brazilian E1 strains (TO-028/BRA, TO-069/BRA, and TO-236/BRA) displayed genetic distance of 0.27–0.61% among each other, closely related to the prototype strains LS451292_ROU/1974 and AF029859_EGY/1951 (17.66–21.04% nt), and to the reference strains identified in Italy and China (0.54–21.34% nt). The E11 TO-086/BRA sequence presented 19.43% of genetic distance compared to the X80059_USA/1953 prototype strain, and 18.09–20.40% when compared to representative E11 group detected in the Caribbean, African, Asian, and North American countries between 2003 and 2017. The two Brazilian E29 (TO-193/BRA and TO-030/BRA) sequences we detected in the present study displayed 20.13–20.88% of genetic distances compared with the AY302552_USA/1958 prototype sequence and 0.29–26.64% compared with the E29 sequences previously detected in Brazil (2014) and Haiti (2016). The TO-018/BRA E3 sequence displayed genetic distance of 22.63% to the prototype AY302553_USA/1951 and ranging from 17.00% to 22.67% nt to references strains from Asia, Europe, and the Caribbean. Genetic distances of 15.15–17.86% nt were observed between TO-032/BRA E30 and the references E30 sequences detected worldwide.

**Figure 2:**
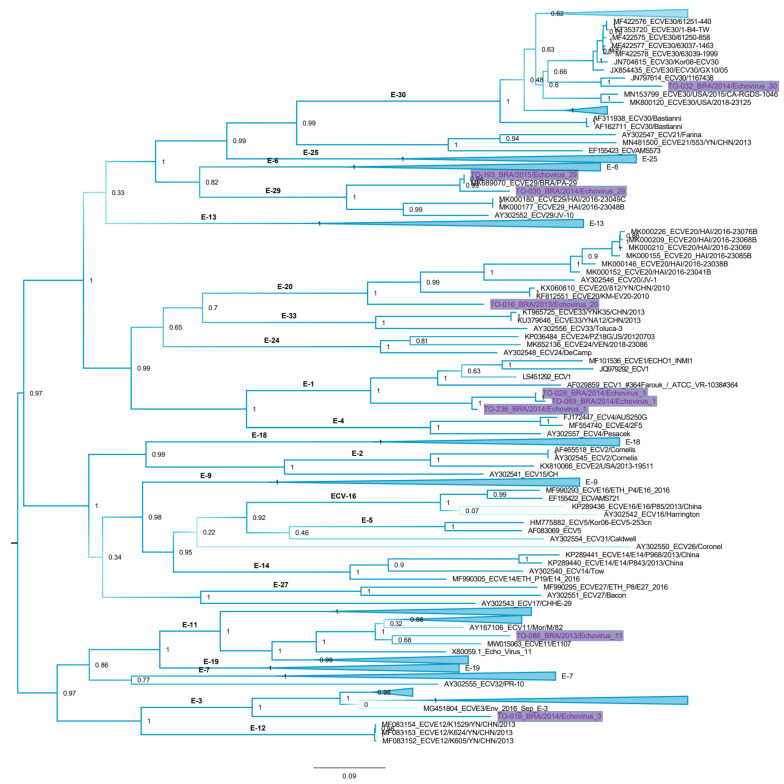
Maximum likelihood tree of VP genome region of enteroviruses. Numbers in each node are the statistical support based on the approximate likelihood test. Echovirus clades are indicated in the tree. Sequences were named following: GenBank ID or number_ country of origin/year of isolation_ genotype. Echoviruses characterized in this study were highlighted in the tree. In order to facilitate visualization, some clusters were collapsed in the tree.

To the phylogenetic inference we have selected a genomic region of enterovirus that is free of recombination. Initially we have estimated the nucleotide similarity per site to determine the regions that have higher variability (Figure S1 upper panel). Next, we used a method to calculate site-by-site recombination rates in the echovirus polyprotein. We found that the nucleotides 1 to 3000 in the alignment (corresponding to the genes VP1, VP 2, VP3, and VP4 of enteriviruses, and here denoted VP region) have the lower rates of recombination (Figure S1 mid panel). Finally, we used likelihood mapping to show that the VP region has a better phylogenetic signal than the remaining regions of the enterovirus genome. This approach shows that in a graphical interface, the percentage of unresolved star-trees in the alignment is in the center of the triangle (Figure S1 lower panel). The higher this percentage, the more inferior the alignment for phylogenetic inferences is. The tree constructed with the VP region of enterovirus shows that the pattern of relatedness of sequences agrees with the serotype classification of Enterovirus Genotyping Tool (Figure 2). The tree also shows the classification of our sequences in six phyloclades (i.e., echovirus 1, echovirus 3, echovirus 11, echovirus 20, vechovirus 29, and echovirus 30). It is interesting to note that Brazilian sequences of echovirus 1 and echovirus 29 form distinct clades and diverge more than 10% from cognate viruses detected elsewhere. For comparative purposes, we also used another approach based on nucleotide similarity to classify sequences ordered according to their similarity in a matrix with frequency distribution of pairwise-identities. Results are in agreement with the phylogenetic analysis and classify our sequences in six groups (Figure S2).

## 4. Discussion

Multiple investigations have described the molecular epidemiology of echovirus globally in both clinical and environmental samples [13,42,43] including in Brazil [16,36]. Nevertheless, there are limited echovirus nucleotide sequences available in the GenBank database [21,44,45,46,47,48], especially full-genome sequences [6].

E1 is an infrequently reported EV genotype in the Western world, accounting for less than 0.1% of all EV genotypes isolated in surveillance studies [7,49,50,51]. It could be speculated that E1 is underreported because patients infected by E1 display only mild symptoms and do not seek medical assistance [52]. On the other hand, E1 is described as a well-known cause of Bornholm disease, a myositis involving muscles of the thorax and upper abdomen [52,53]. In Brazil, E1 has been associated with aseptic meningitis [49]. Until now, there have been no E1 nucleotide sequences from Brazil available in the GenBank database, and virtually no phylogenetic studies evaluated this genotype. Our phylogenetic analysis showed E1 in Brazil is divergent and form a distinct phyloclade.

E3 leads to a broad spectrum of clinical manifestations [54,55]. It is a globally dispersed but rarely reported echovirus genotype and displays low rates of prevalence [7,54,56,57,58,59]. It has been suggested that E3 exhibits a distinct epidemic pattern of circulation with regular increases every four to seven years [7,60]. In recent years, E3 has been identified with increased frequency in Brazil, although maintaining its minor pattern of prevalence [17,46,47,48]. Sequencing methods have become more accessible, and genetic characterization of EV strains is the basis for current EV genotyping [60], perhaps leading to an increased E3 identification. Continuous surveillance of E3 is vital to provide further information on its circulation in this country.

E11 is one of the most commonly isolated human enteroviruses [59,61], frequently associated with neonatal systemic illness [62,63] and displays an epidemic pattern of circulation [7,61]. The majority of E11 detection occur in infants aged <1 year [63], as observed here. E11 was detected in 6.7% of the stool samples analyzed from symptomatic acute flaccid paralysis patients in Brazil [48], and a regular distribution of its association with aseptic meningitis cases has also been reported [43,46], suggesting a high occurrence of this genotype in this country. Phylogenetic analysis of the VP1 gene has indicated the existence of multiple E11 lineages and/or subtypes [61]. Although a common genotype, limited E11 sequences are available from Brazil. A comprehensive study of E11 molecular epidemiology in Brazil is needed. As Brazil is a vast country, E11 strains circulating in each region may exhibit distinct characteristics.

Historically, E20 has been very rare in most surveillance system reports [7,57,58,64], including in Brazil [48]. E20 shows a tendency to recur at variable intervals [7]. This cyclic occurrence suggests that the E20 genotype circulates over a period of time in a country or region followed by development of highly specific immunization of the population. A subsequent period of an interruption in virus circulation leads to a build-up of susceptible individuals, resulting in re-introduction of the virus and its transmission [59]. The E20 detection reported here, together with previous findings, underlines the importance of intensifying non-polio EV monitoring in order to understand the etiological role of E20 in Brazil.

Despite E29 being detected globally, epidemiological data on EV from Europe, Americas, Asia, and Africa reveal that this particular genotype exhibits low rates of frequency [6,48,56,59,65,66,67,68], corroborating its rarity in the circulation. The reason for this particular pattern of circulation is still unclear but is frequently also observed for other echovirus strains [7,60]. Although traditional neutralization antisera and molecular methods are currently available for E29 identification and characterization [69,70], its rare pattern of detection seems to impair genetic studies. Only a few E29 complete genome sequences are available in the GenBank database, and there are practically no genomic studies addressing this genotype [6]. E29 sequences described in our study form a distinct phylogenetic clade compared with E29 strains.

E30 consistently appeared among the most commonly reported EV worldwide, particularly associated with aseptic meningitis, including in Brazil [7,43,46]. Waves of E30 activity are associated with distinct new genomic lineages, which usually replace ones that were previously circulating [8,71,72]. A continuous circulation of E30 in Brazil has been observed since 1998, and VP1 gene phylogenetic analysis suggests that distinct E30 introductions occurred in this country [21]. The vast Brazilian territory associated with recurrent E30 detection throughout the decades [21,43,44,46,49] highlights the importance of continuous EV clinical surveillance.

The full-genome analysis conducted here, with the exception of E11 and E30 genotypes, were limited by the availability of complete sequences of the rarely identified genotypes (i.e., E1, E3, E20, and E29). However, the genetic relationship between echovirus genotypes characterized in the present investigation and contemporary strains from distant geographical regions highlight the ability of EV to spread over large geographical areas. This emphasizes the importance of EV surveillance worldwide [60]. The data on echovirus genotypes circulating in the Northern region of Brazil and their genomic identity contributes to our knowledge of echovirus diversity circulating in this country, as well as providing a baseline for future molecular analysis studies.

It is important to mention that the echovirus sequences detected here were identified in fecal specimens during a stool sample survey of children suffering from acute gastroenteritis. Echoviruses are classically not known to be associated with acute diarrhea [7,60], and the gastroenteritis symptoms observed in these patients were most probably linked to one (or more) of the gastroenteric viruses detected in their fecal samples (i.e., norovirus and rotavirus) (see the amount of all viruses identified in these individuals in Figure S3).

In summary, this study adds new information on echovirus genetic diversity in Brazil, a country where data on viral sequencing are still scarce. NGS surveillance applied in the present investigation to study enteric viruses has led to identification of rarely reported echovirus strains (i.e., E1, E3, E20, and E29). A multiregional surveillance is vital to establish a more complete epidemiological-molecular picture of the EV burden in Brazil. Identifying circulating echoviruses can help to elucidate enteroviral biodiversity, improve our understanding of their potential health burden, and enable a prompt response in case of outbreaks. This is especially valuable considering the capacity of EV to remain in asymptomatic circulation in populations [6]. The data obtained here together with recent reports [17,43,46,47] reinforce the urgent need to establish an effective non-polio EV surveillance system in Brazil.

## Figures and Tables

**Table 1:** Socio-demographic and molecular data of positive echovirus samples from patients with acute gastroenteritis in Northern Brazil.

Age (Months)	Gender	Municipality	State	Year	Genotype	Strain	Symptoms	Co-Infections (Viruses)	Length (nt)	BLAST Score	Accession Numbers
25	F	Paraíso do Tocantins	TO	2013	E20	TO-016	Diarrhea	RVA/NoV/E99	7339	80%	MW775340
18	M	Araguaína	TO	2014	E3	TO-018	Diarrhea	RVA/HAdV	7264	81%	MW775341
7	M	Araguaína	TO	2014	E1	TO-028	Diarrhea	RVA/HAdV	7401	79%	MW775342
50	F	Araguaína	TO	2014	E29	TO-030	Diarrhea	RVA/HAdV/PeV	7230	79%	MW775343
8	M	Araguaína	TO	2014	E30	TO-032	Diarrhea, vomiting and fever	NoV/HAdV/HAstV	7562	83%	MW775344
11	M	Goiatins	TO	2014	E1	TO-069	Diarrhea and fever	RVA/HAdV	7418	79%	MW775345
9	M	Araguaína	TO	2013	E11	TO-086	Diarrhea, vomiting and fever	RVA/NoV/HAdV	7427	82%	MW775346
15	F	Araguaína	TO	2015	E29	TO-193	Diarrhea	SAFV	7412	80%	MW775347
12	M	Araguaína	TO	2014	E1	TO-236	Diarrhea vomiting and fever	RVA	7375	77%	MW775348

State of Tocantins (TO), Rotavirus group A (RVA), Norovirus (NoV), Enteric human adenovirus (HAdV), human parechoviruses (Pev), Saffold virus (SAFV).

## Data Availability

Not applicable.

## Supplementary Materials

The following are available online at https://www.mdpi.com/article/10.3390/v13040595/s1, Figure S1: Nucleotide variability of echovirus polyprotein, Figure S2: Nucleotide similarity matrix, Figure S3: Amount of viruses in samples, Table S1: List of reference sequences.
