# Supplementary material for: High Heterogeneity of Echoviruses in Brazilian Children with Acute Gastroenteritis
Source: Viruses. 2021 Mar 31;13(4):595. doi: 10.3390/v13040595 (PMC8067319; doi:10.3390/v13040595)
Supplement: Supplementary file 1 [file viruses-13-00595-s001.pdf]

# Supplementary material

## High heterogeneity of Echoviruses in Brazilian children with acute gastroenteritis

Endrya do Socorro Fôro Ramos1, Ulisses Alves Rosa1, Geovani de Oliveira Ribeiro1, Fabiola Villanova1, Flávio Augusto de Pádua Milagres2,3, Rafael Brustulin2, Vanessa dos Santos Morais4, Mayara Bertanhe4, Roberta Marcatti4, Emerson Luiz Lima Araújo5, Steven S. Witkin4,6, Eric Delwart7,8, Adriana Luchs9,*, Antonio Charlys da Costa4,* and Élcio Leal1,*

1 Laboratório de Diversidade Viral, Instituto de Ciências Biológicas, Universidade Federal do Pará, Belem, Pará, Brazil;66075-000, Brazil; endrya.ramos@gmail.com (E.R); ualvesfisio@yahoo.com.br (U.A.R); geovanibiotec@gmail.com (G.O.R); elcioleal@gmail.com (E.L.); fvillanova@gmail.com (F.V.)
2 Secretaria de Saúde do Tocantins, Tocantins 77453-000, Brazil; eu3rafael@gmail.com (R.B.); flaviomilagres@uft.edu.br (F.A.d.P.M)
3 Laboratório Central de Saúde Pública do Tocantins (LACEN/TO), Tocantins 77016-330, Brazil
4 Departamento de Moléstias Infecciosas e Parasitárias and Instituto de Medicina Tropical da Faculdade de Medicina da Universidade de São Paulo, São Paulo, Brazil; va.morais@usp.br (V.S.M); mayarabertanhe@globo.com (M.B); robertamarcatti@gmail.com (R.M); charlysbr@yahoo.com.br (A.C.d.C)
5 General Coordination of Public Health Laboratories of the Strategic Articulation Department of the Health Surveillance Secretariat of the Ministry of Health (CGLAB/DAEVS/SVS-MS), 70719-040, Brasília, DF, Brazil; emerson.araujo@saude.gov.br (E.L.L.A)
6 Department of Obstetrics and Gynecology, Weill Cornell Medicine, New York, NY, USA;1300 York Avenue New York, NY 10065 USA; switkin@med.cornell.edu (S.S.W.)
7 Vitalant Research Institute, 270 Masonic Avenue, San Francisco, USA;San Francisco, 94143 CA, USA; EDelwart@Vitalant.org (E.D)
8 Department Laboratory Medicine, University of California San Francisco, San Francisco, CA, 94143, USA.
9 Laboratório de Doenças Entéricas, Centro de Virologia, Instituto Adolfo Lutz, São Paulo 01246-000, Brazil; driluchs@gmail.com (A.L.)
* Correspondence: charlysbr@yahoo.com.br (A.C.d.C); elcioleal@gmail.com (É.L.)

Figure S1

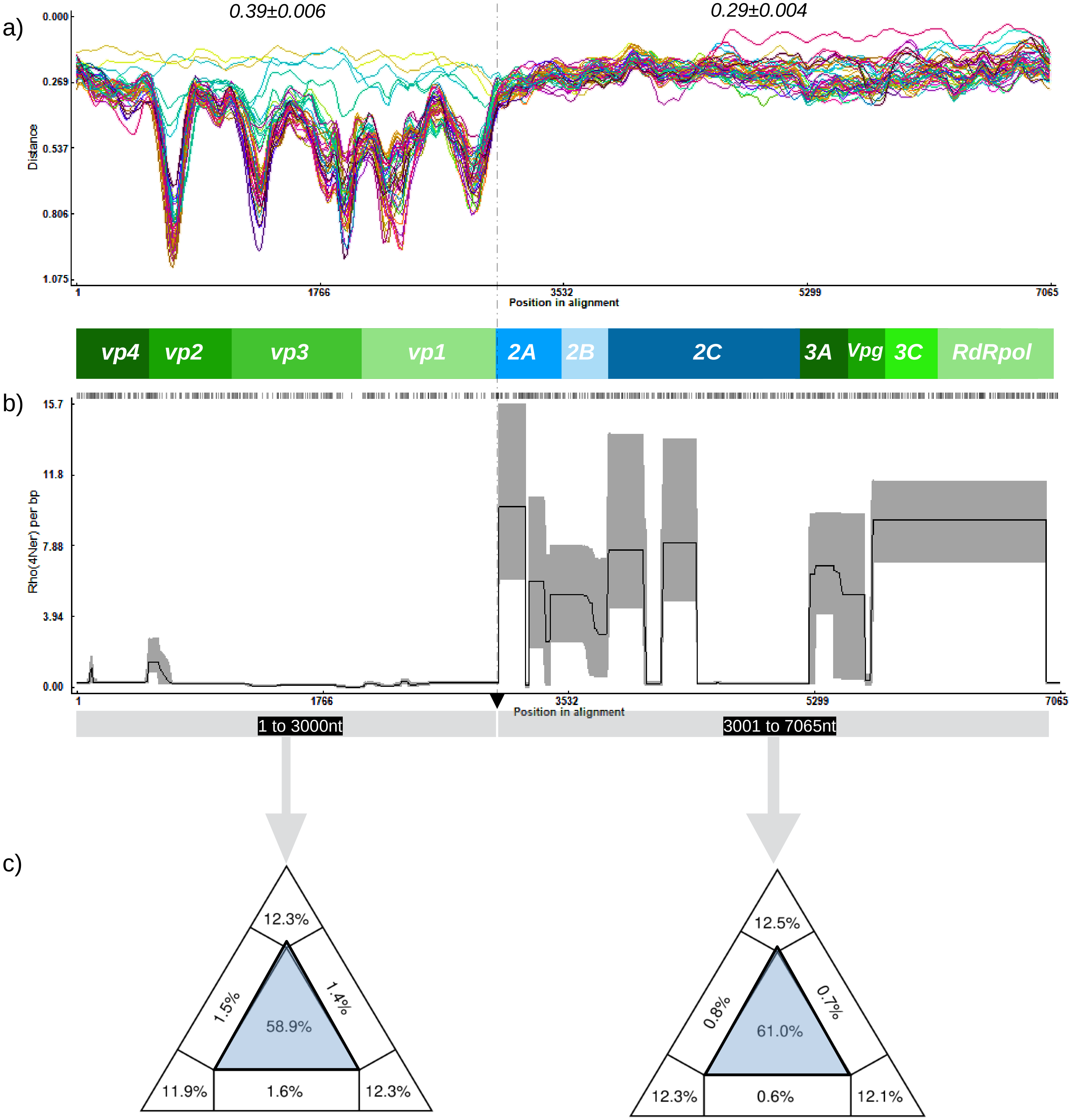


Figure S1. Nucleotide variability of echovirus polyprotein region. A) Nucleotide similarity plot of polyprotein genomic region of echoviruses. Each line represent one sequence included in this analysis (see Table S1 for the description of references sequences used). In the x-axis lines represent pair wise distances of each site in the alignment. The y-axis indicate the nucleotide position in the alignment. The vertical line indicate the position which the alignment was partitioned. Number above the lines are the mean genetic distances and their standard errors calculated in each partition. B) Recombination rates in the echovirus polyprotein. The dark line represents the calculated site-by-site recombination rate in the echovirus polyprotein and the gray area is the 95% credibility interval. Between a) and b) there is a diagram showing the genes of the echovirus polyprotein. c) Likelihood mapping of each partition. This approach shows the percentage of unresolved star-trees in the alignment in the center of the triangle. The higher this percentage, the more inferior is the alignment for phylogenetic inferences.

# Figure S2

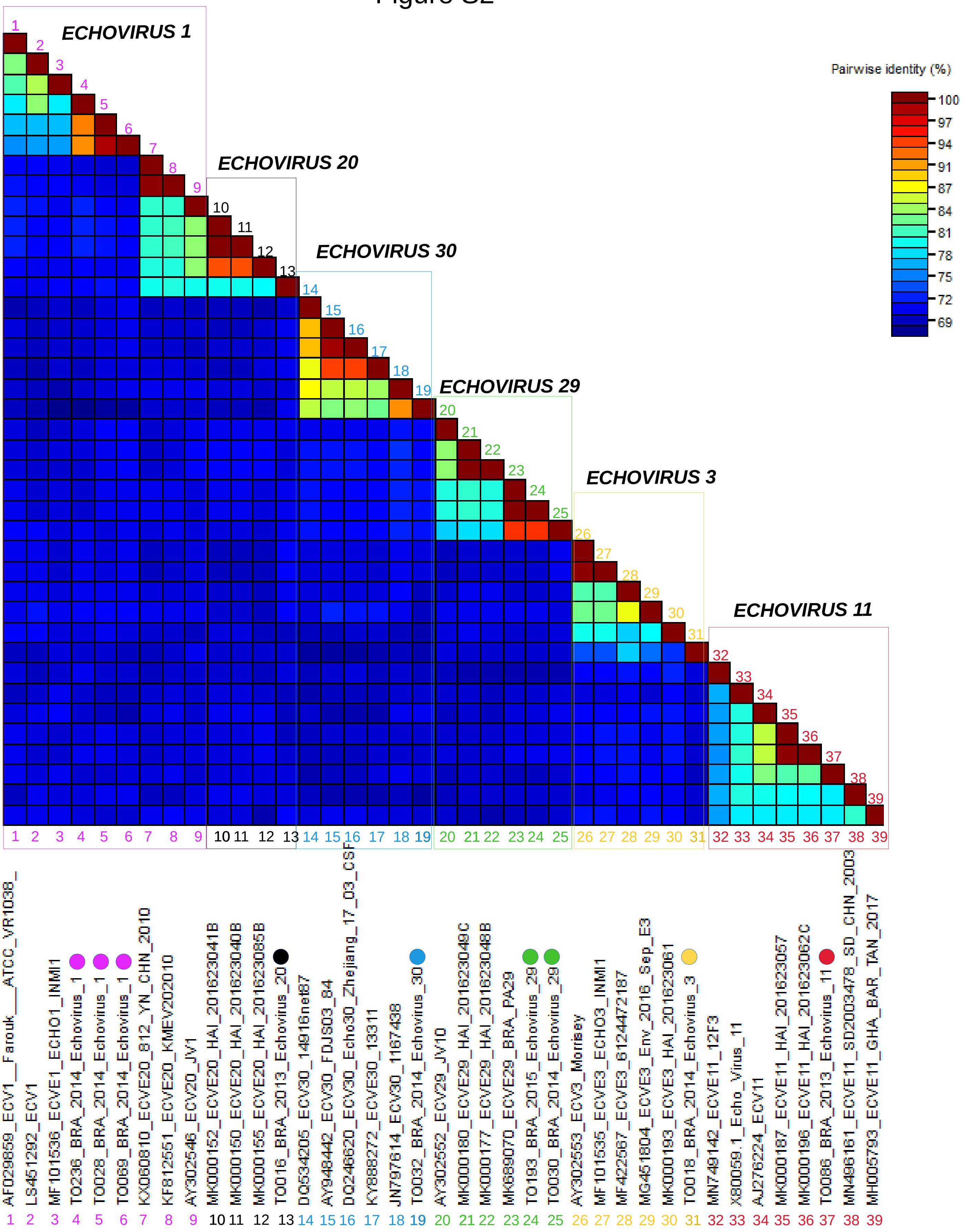


Figure S2. Nucleotide similarity matrix of echoviruses. The similarity of all pairs of sequences are indicated in colors according to the scale in the figure. Echovirus serotypes are indicated by colored rectangles (Magenta =serotype 1; black=serotype 20; light blue= serotype 30; gree= serotype 29; yellow=seroptype 3 and red=serotype 11). Sequences generated in this study are indicated by colored circles.

# Figure S3

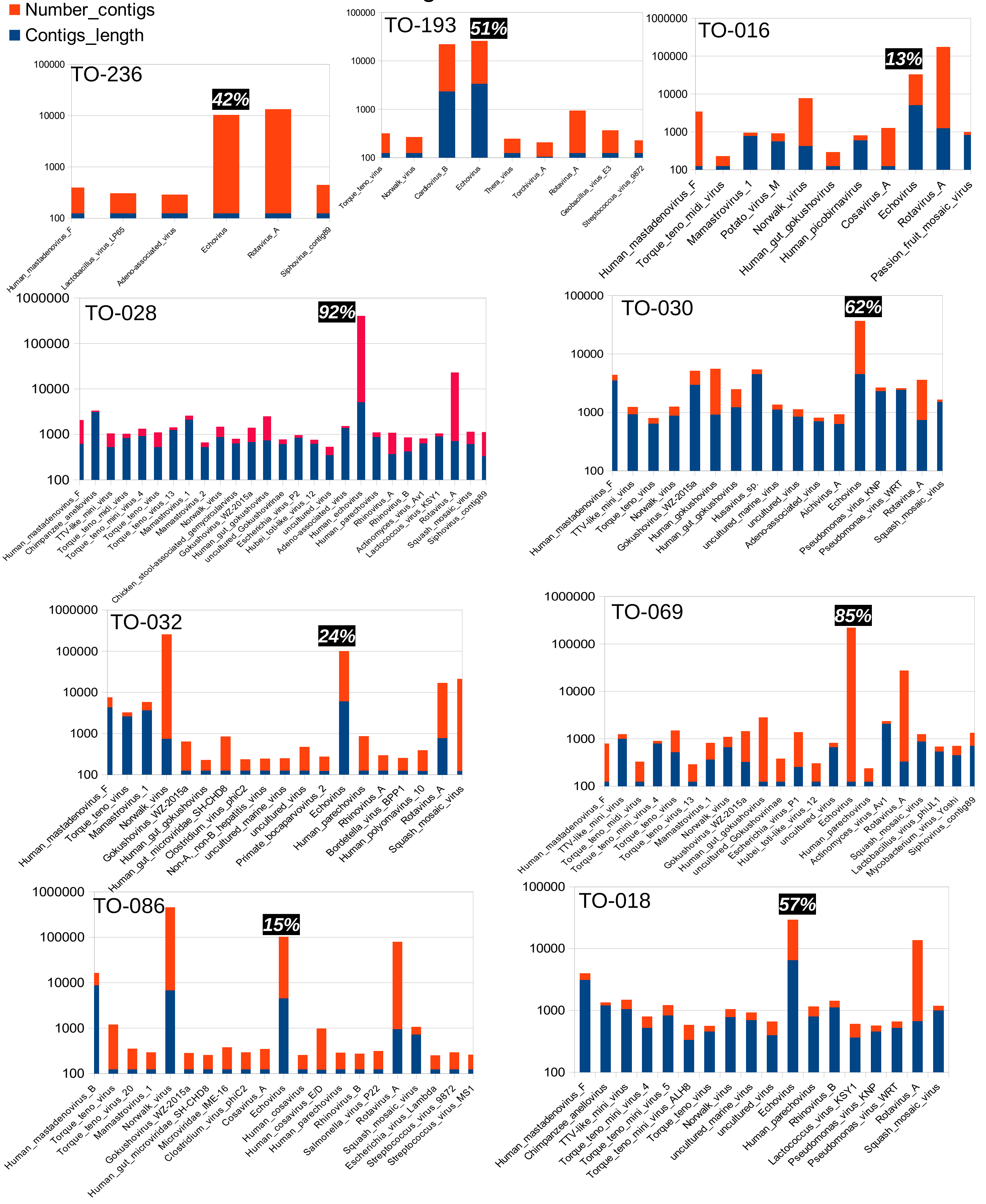


Figure S3. Amount of other viruses present in samples where Echoviruses were detected in this study. The y-axis is in log scale and show the number of reads (red) and contig length (blue) of viruses detected in each sample. Numbers in the dark rectangles are the percentage of echovirus contigs detected in each sample.

# Table S1 List of 351 reference sequences in this study

| Genbank ID | Serotype/Sequence name |
|---|---|
| AY036579 | ECV7/Wallace |
| AY036578 | ECV7/UMMC |
| AB501332 | ECVE13 |
| AB501331 | ECVE13 |
| AF317694 | ECV18/Metcalf |
| MF101536 | ECVE1/ECHO1_INMI1 |
| MF101535 | ECVE3/ECHO3_INMI1 |
| KX139460 | ECVE25/Jena/VI10308/10 |
| KX139459 | ECVE25/Jena/AN1380/10 |
| KX139458 | ECVE18/Jena/AN1389/10 |
| KX139457 | ECVE18/Jena/AN1384/10 |
| KX139456 | ECVE18/Jena/AN1371/10 |
| KX139455 | ECVE18/Jena/AN1370/10 |
| KX139454 | ECVE18/Jena/AN1367/10 |
| KX139453 | ECVE18/Jena/AN1366/10 |
| KX139452 | ECVE18/Jena/AN1365/10 |
| KX139451 | ECVE18/Jena/AN1363/10 |
| KX139450 | ECVE18/Jena/AN1362/10 |
| KX139449 | ECVE18/Jena/VI10517/10 |
| KX139448 | ECVE18/Jena/VI10245/10 |
| KX139447 | ECVE18/Jena/VI10227/10 |
| KX139446 | ECVE18/Jena/ST9524/10 |
| AY167107 | ECV19/K/542/81 |
| AY167106 | ECV11/Mor/M/82 |
| AY167105 | ECV11/Kust/86 |
| AY167104 | ECV11/Kar/87 |
| AY167103 | ECV11/Hun/90 |
| EF066392 | ECV30/TW/3182/01 |
| EF066391 | ECV30/TW/2513/01 |
| JN797614 | ECV30/1167438 |
| MN597954 | ECVE11/Sewage6-1-1/GD.GZ/CHN/2019 |
| MN597953 | ECVE11/Sewage15-2-1/GD.GZ/CHN/2019 |
| MN597952 | ECVE11/HFMD1311-GD-CHN-2018 |
| MN597951 | ECVE11/Sewage4-2-1/GD.GZ/CHN/2019 |
| MN597950 | ECVE11/Sewage34-1-1/GD.GZ/CHN/2018 |
| MN153799 | ECVE30/USA/2015/CA-RGDS-1046 |
| KP294524 | ECVE11/520k/YN/CHN/2010 |
| AB501330 | ECVE13 |
| AB501329 | ECVE13 |
| MW015064 | ECVE11/E1108 |
| MW015063 | ECVE11/E1107 |
| MW015062 | ECVE11/E1106 |
| MW015061 | ECVE11/E1105 |
| MW015060 | ECVE11/E1104 |
| MW015059 | ECVE11/E1103 |
| MW015058 | ECVE11/E1102 |
| MW015057 | ECVE11/E1101 |
| MT350224 | ECVE18/E18/JXY2-2/2019 |
| MN832718 | ECVE18/QD10/SD/CHN/2019 |
| MN832717 | ECVE18/QD9/SD/CHN/2019 |
| MN737190 | ECVE18/QD5-C/SD/CHN/2019 |
| MN737188 | ECVE18/QD7/SD/CHN/2019 |
| MN737187 | ECVE18/QD6/SD/CHN/2019 |
| MN737186 | ECVE18/QD5/SD/CHN/2019 |
| MN737185 | ECVE18/QD4/SD/CHN/2019 |
| MK000232 | ECVE11/HAI/2017-23083B |
| MK000231 | ECVE7/HAI/2017-23083A |
| MK000230 | ECVE13/HAI/2017-23082B |
| MK000229 | ECVE7/HAI/2017-23082A |
| MK000227 | ECVE6/HAI/2016-23077A |
| MK000226 | ECVE20/HAI/2016-23076B |
| MK000224 | ECVE13/HAI/2016-23075 |
| MK000223 | ECVE7/HAI/2016-23074 |
| MK000222 | ECVE13/HAI/2016-23073 |
| MK000221 | ECVE13/HAI/2016-23072 |
| MK000219 | ECVE19/HAI/2017-23081A |
| MK000217 | ECVE6/HAI/2017-23080A |
| MK000216 | ECVE19/HAI/2017-23079 |
| MK000215 | ECVE13/HAI/2017-23078B |
| MK000213 | ECVE11/HAI/2016-23071 |
| MK000212 | ECVE19/HAI/2016-23070B |
| MK000211 | ECVE11/HAI/2016-23070A |
| MK000210 | ECVE20/HAI/2016-23069 |
| MK000209 | ECVE20/HAI/2016-23068B |
| MK000208 | ECVE6/HAI/2016-23068A |
| MK000207 | ECVE19/HAI/2016-23067B |
| MK000206 | ECVE3/HAI/2016-23067A |
| MK000203 | ECVE11/HAI/2016-23065B |
| MK000201 | ECVE19/HAI/2016-23064B |
| MK000200 | ECVE11/HAI/2016-23064A |
| MK000199 | ECVE19/HAI/2016-23063B |
| MK000198 | ECVE6/HAI/2016-23063A |
| MK000197 | ECVE19/HAI/2016-23062D |
| MK000196 | ECVE11/HAI/2016-23062C |

| Genbank ID | Serotype/Sequence name |
|---|---|
| MK000193 | ECVE3/HAI/2016-23061 |
| MK000192 | ECVE19/HAI/2016-23053 |
| MK000191 | ECVE19/HAI/2016-23052 |
| MK000190 | ECVE11/HAI/2016-23060 |
| MK000189 | ECVE6/HAI/2016-23059 |
| MK000188 | ECVE6/HAI/2016-23058 |
| MK000187 | ECVE11/HAI/2016-23057 |
| MK000186 | ECVE3/HAI/2016-23056 |
| MK000185 | ECVE6/HAI/2016-23055 |
| MK000184 | ECVE19/HAI/2016-23054 |
| MK000183 | ECVE6/HAI/2016-23051B |
| MK000182 | ECVE3/HAI/2016-23051A |
| MK000180 | ECVE29/HAI/2016-23049C |
| MK000179 | ECVE7/HAI/2016-23049B |
| MK000178 | ECVE6/HAI/2016-23049A |
| MK000175 | ECVE19/HAI/2016-23047 |
| MK000172 | ECVE6/HAI/2016-23045 |
| MK000171 | ECVE6/HAI/2016-23044 |
| MK000170 | ECVE6/HAI/2016-23043 |
| MK000169 | ECVE19/HAI/2016-23042B |
| MK000168 | ECVE6/HAI/2016-23042A |
| MK000167 | ECVE6/HAI/2016-23035 |
| MK000166 | ECVE6/HAI/2016-23034 |
| MK000165 | ECVE19/HAI/2016-23037E |
| MK000164 | ECVE19/HAI/2016-23037D |
| MK000162 | ECVE6/HAI/2016-23037B |
| MK000154 | ECVE6/HAI/2016-23085A |
| MK000153 | ECVE7/HAI/2016-23084 |
| MK000152 | ECVE20/HAI/2016-23041B |
| MK000149 | ECVE19/HAI/2016-23040A |
| MK000148 | ECVE19/HAI/2016-23039B |
| MK000146 | ECVE20/HAI/2016-23038B |
| MH484075 | ECVE30/20L |
| MH484074 | ECVE30/19L |
| MH484073 | ECVE30/18L |
| MH484072 | ECVE30/15L |
| MF990305 | ECVE14/ETH_P19/E14_2016 |
| MF990301 | ECVE18/ETH_P12/E18_2016 |
| MF990295 | ECVE27/ETH_P8/E27_2016 |
| MF990293 | ECVE16/ETH_P4/E16_2016 |
| MF990292 | ECVE19/ETH_P3/E19_2016 |
| MH005794 | ECVE6/GHA#136CEN#136UDW/2017 |
| MH005793 | ECVE11/GHA#137BAR#137TAN/2017 |
| MH005792 | ECVE13/GHA#138VOL#138KRN/2017 |
| MH005791 | ECVE6/GHA#139CEN#139ASE/2017 |
| MH005790 | ECVE11/GHA#140UER#140PUS/2017 |
| MG571850 | ECVE7 |
| MG571848 | ECVE7 |
| MF422580 | ECVE9/61253-70985 |
| MF422579 | ECVE6/61252-70738 |
| MF422578 | ECVE30/63039-1999 |
| MF422577 | ECVE30/63037-1463 |
| MF422576 | ECVE30/61251-440 |
| MF422575 | ECVE30/61250-858 |
| MF422572 | ECVE3/63040-70881 |
| MF422570 | ECVE3/61247-622 |
| MF422569 | ECVE3/61246-70294 |
| MF422567 | ECVE3/61244-72187 |
| MF422565 | ECVE25/61242-1563 |
| MF422564 | ECVE25/61241-70868 |
| EF155423 | ECVAMS573 |
| EF155422 | ECVAMS721 |
| X92886 | ECV9 |
| AJ577594 | ECV11 ROU-9191 |
| AJ577590 | ECV11FIN-0666 |
| AJ577589 | ECV11/HUN-1108 |
| MH745407 | ECVE19/NGR_2014 |
| DQ534205 | ECV30/14916net87 |
| MK791152 | ECVE11/2017-122-R2 |
| MK791151 | ECVE6/78R2 |
| MK791150 | ECVE3/123-R2 |
| MH732737 | ECVE7/Ibadan_NGR_2010 |
| KX641241 | ECVE6/K843/YN/CHN/2013 |
| KX641240 | ECVE6/K727/YN/CHN/2013 |
| KY792585 | ECVE19/PDV_BLR_IN |
| KX060810 | ECVE20/812/YN/CHN/2010 |
| KP036484 | ECVE24/PZ18G/JS/20120703 |
| KJ765699 | ECVE7/DH22G/JS/2012 |
| JQ801739 | ECVE6/KM57-09 |
| JN596587 | ECVE9/MSH/KM812/2010 |
| AF465518 | ECV2/Cornelis |
| AF465517 | ECV6/Charles |
| AF465516 | ECV7/Wallace |
| JN704615 | ECV30/Kor08-ECV30 |

| Genbank ID | Serotype/Sequence name |
|---|---|
| HM775882 | ECV5/Kor06-ECV5-253cn |
| AF311938 | ECV30/Bastianni |
| MK689070 | ECVE29/BRA/PA-29 |
| MN657230 | ECVE11/E11-NHP-RD31 |
| MH830353 | ECVE6/E6-366/HB/CHN/2015 |
| KT965725 | ECVE33/YNK35/CHN/2013 |
| AB647326 | ECVE3/OC10-798 |
| AB647325 | ECVE3/OC10-774 |
| AB647324 | ECVE3/OC10-731 |
| AB647323 | ECVE3/OC10-571 |
| AB647322 | ECVE3/OC10-540 |
| AB647321 | ECVE3/OC10-532 |
| AB647320 | ECVE3/OC10-492 |
| AB647319 | ECVE3/OC10-487 |
| AB647318 | ECVE3/OC10-478 |
| AB647317 | ECVE3/OC10-448 |
| AB647316 | ECVE3/OC10-380 |
| KU379646 | ECVE33/YNA12/CHN/2013 |
| KY828852 | ECVE18/A86/YN/CHN/2016 |
| KY828851 | ECVE18/A83/YN/CHN/2016 |
| KX619440 | ECVE6/RA/E6/Ahvaz/Iran/2011 |
| JX976773 | ECVE30/E30SD2010CHN |
| JX976772 | ECVE25/E25SD2010CHN |
| JX976771 | ECVE6/E6SD11CHN |
| AB705311 | ECVE6 |
| AB705310 | ECVE6 |
| AB705308 | ECVE6 |
| HM031191 | ECV25/HN-2 |
| HM777023 | ECV18/Kor05-ECV18-054cn |
| AF524867 | ECV9/DM |
| AF524866 | ECV9/Barty |
| DQ246620 | ECV30/Echo30/Zhejiang/17/03/CSF |
| AF162711 | ECV30/Bastianni |
| AF083069 | ECV5 |
| AY302559 | ECV7/Wallace |
| AY302558 | ECV6/D'Amori |
| AY302557 | ECV4/Pesacek |
| AY302556 | ECV33/Toluca-3 |
| AY302555 | ECV32/PR-10 |
| AY302554 | ECV31/Caldwell |
| AY302553 | ECV3/Morrisey |
| AY302552 | ECV29/JV-10 |
| AY302551 | ECV27/Bacon |
| AY302550 | ECV26/Coronel |
| AY302549 | ECV25/JV-4 |
| AY302548 | ECV24/DeCamp |
| AY302547 | ECV21/Farina |
| AY302546 | ECV20/JV-1 |
| AY302545 | ECV2/Cornelis |
| AY302544 | ECV19/Burke |
| AY302543 | ECV17/CHHE-29 |
| AY302542 | ECV16/Harrington |
| AY302541 | ECV15/CH |
| AY302540 | ECV14/Tow |
| AY302539 | ECV13/Del |
| FJ172447 | ECV4/AUS250G |
| U16283 | ECV6 |
| EF634316 | ECV11/D207 |
| X84981 | ECV9 |
| KT353720 | ECVE30/1-B4-TW |
| MN749146 | ECVE18/12J3 |
| MN749143 | ECVE18/12G5 |
| MN166092 | ECVE18/USA/2015/CA-RGDS-1049 |
| MF678320 | ECVE9/NSW-V32-2008-ECHO9 |
| MF678316 | ECVE7/NSW-V28-2007-ECHO7 |
| MF678296 | ECVE25/NSW-V07-2007-ECHO25 |
| MF554740 | ECVE4/2F5 |
| KX808644 | ECVE3/JSev001 |
| KJ957190 | ECVE25/E25/2010/CHN/BJ |
| KP266571 | ECVE30/2002-59 |
| KP266570 | ECVE7/2001-31 |
| KP266568 | ECVE6/2005-29-1 |
| KC897073 | ECVE30/2012EM161 |
| KF042343 | ECVE6/2012EM100 |
| KF042342 | ECVE6/2012EM95 |
| JQ929657 | ECVE6/FIN09-NPA |
| JX854435 | ECVE30/ECV30/GX10/05 |
| JQ729993 | ECV6/FJLY2010327 |
| HM185056 | ECV6/Echo6/Henan/127/2008 |
| HM185055 | ECV6/Echo6/Henan/116/2008 |
| MW080377 | ECVE30/TL7C/NM/CHN/2016 |
| MW080372 | ECVE30/TL12C/NM/CHN/2016 |
| MN215884 | ECVE18/LJ/0530/2019 |
| MN496161 | ECVE11/SD2003-478/SD/CHN/2003 |

# Table S1 List of 351 reference sequences in this study (cont.)

| Genbank ID | Serotype/Sequence name |
|---|---|
| MH043137 | ECVE7/RIGVIR |
| MH043136 | ECVE7/07VI447 |
| MH043135 | ECVE7/98-60628 |
| MH043134 | ECVE7/98-59065 |
| MH043133 | ECVE7/98-57213 |
| MH043132 | ECVE7/Wallace |
| MG720261 | ECVE18/E18-HeB15-54498/HeB/CHN/2015 |
| MG720260 | ECVE18/E18-HeB15-54462/HeB/CHN/2015 |
| MG720259 | ECVE18/E18-398/HeB/CHN/2015 |
| MG720258 | ECVE18/E18-393/HeB/CHN/2015 |
| MG720257 | ECVE18/E18-291/HeB/CHN/2015 |
| MG720256 | ECVE18/E18-221/HeB/CHN/2015 |
| MK532311 | ECVE25/USA/CA/RGDS-2017-1010 |
| MK580458 | ECVE9/USA/CA/RGDS-2017-1011 |
| MK238483 | ECVE30/USA/2017/CA-RGDS-1005 |
| MF083154 | ECVE12/K1529/YN/CHN/2013 |
| MF083153 | ECVE12/K624/YN/CHN/2013 |
| MF083152 | ECVE12/K605/YN/CHN/2013 |
| KY888274 | ECVE30/14-397 |
| KY888273 | ECVE30/13-759 |
| KY888272 | ECVE30/13-311 |
| KY981581 | ECVE11/9368/ISR/1998 |
| KY981580 | ECVE11/9310/ISR/1998 |
| KY981579 | ECVE11/9295/ISR/1998 |
| KY981578 | ECVE11/8640/ISR/1998 |
| KY981577 | ECVE11/8416/ISR/1997 |
| KY981576 | ECVE11/8108/ISR/1996 |
| KY981575 | ECVE11/8098/ISR/1996 |
| KY981574 | ECVE11/7676/ISR/1996 |
| KY981573 | ECVE11/7482/ISR/1995 |
| KY981572 | ECVE11/6067/ISR/1993 |
| KY981571 | ECVE11/5960/ISR/1993 |
| KY981570 | ECVE11/5824/ISR/1992 |
| KY981569 | ECVE11/5789/ISR/1992 |
| KY981568 | ECVE11/1541/ISR/1999 |
| KY981567 | ECVE11/1513/ISR/1999 |
| KY981566 | ECVE11/1512/ISR/1999 |
| KY981565 | ECVE11/1510/ISR/1999 |
| KY981564 | ECVE11/1373/ISR/1999 |
| KY981563 | ECVE11/1315/ISR/1999 |
| KY981562 | ECVE11/1096/ISR/1999 |
| KY981561 | ECVE11/1000/ISR/1999 |
| KY981560 | ECVE11/675/ISR/1999 |
| KY981559 | ECVE11/674/ISR/1999 |
| KY981558 | ECVE11/535/ISR/1999 |
| KY981557 | ECVE11/23/ISR/1997 |
| KX774483 | ECVE25/E25/ZE-wly/Zhejiang/CHN/2005 |
| KX527626 | ECVE11/ISO_VR_Echo11 |
| KX767786 | ECVE18/E18-314/HB/CHN/2015 |
| KM388538 | ECVE3/HNWY-01 |
| KM024043 | ECV3/Morrisey |
| KP099941 | ECVE25/XM0297 |
| KP202389 | ECVE7/3988/09 |
| KF812551 | ECVE20/KM-EV20-2010 |
| KC238668 | ECVE9/DM34 |
| KC238667 | ECVE9/DM33 |
| MN481500 | ECVE21/553/YN/CHN/2013 |
| KT353725 | ECVE6/2-D5-TW |
| KT353724 | ECVE6/2-C2 |
| KT353723 | ECVE3/2-E6-TW |
| MK800120 | ECVE30/USA/2018-23125 |
| MT347976 | ECVE25/USA/2016-19521 |

| Genbank ID | Serotype/Sequence name |
|---|---|
| MN749159 | ECVE11/10S1 |
| MN749142 | ECVE11/12F3 |
| MN145871 | ECVE6/EchoE6/22/ZJ/CHN/2018 |
| LC416536 | ECVE30 |
| LC416535 | ECVE30 |
| LC416534 | ECVE30 |
| LC416533 | ECVE30 |
| MK652137 | ECVE11/USA/2018-23090 |
| MK652136 | ECVE24/VEN/2018-23086 |
| MK800121 | ECVE25/USA/2018-23126 |
| MH144605 | ECVE9/11-2042-1 |
| MH118026 | ECVE25/10-4339-2 |
| MK159694 | ECVE7/Nigeria/AFP/2014 |
| MH752989 | ECVE11/USA/MI/2016-23031 |
| MH752986 | ECVE9/USA/MI/2004-23028 |
| MG451810 | ECVE7/Env_2017_Jan_E-7 |
| MG451806 | ECVE7/Env_2016_Sep_E-7b |
| MG451805 | ECVE7/Env_2016_Sep_E-7a |
| MG451804 | ECVE3/Env_2016_Sep_E-3 |
| KX610685 | ECVE9/UW1 |
| KY645964 | ECVE30/16-I10 |
| KX810066 | ECVE2/USA/2013-19511 |
| KX681481 | ECVE9/UW0 |
| KU355273 | ECVE7/40/Longyou/ZJ |
| KP289441 | ECVE14/E14/P968/2013/China |
| KP289440 | ECVE14/E14/P843/2013/China |
| KP289439 | ECVE6/E6/P735/2013/China |
| KP289436 | ECVE16/E16/P85/2013/China |
| AF029859 | ECV1_#364Farouk_/_ATCC_VR-1038#364 |
| LS451292 | ECV1 |
| X80059 | ECV11 |
| AJ276224 | ECV11 |
| MK000155 | ECVE20_HAI/2016-23085B |
| AY948442 | ECV30_FDJS03_84 |
| KF878942 | ECVE30_KM/A363/09 |
| MK000177 | ECVE29_HAI/2016-23048B |
| JQ979292 | ECV1 |
